# Expression of SIRT1 and DBC1 Is Associated with Poor Prognosis of Soft Tissue Sarcomas

**DOI:** 10.1371/journal.pone.0074738

**Published:** 2013-09-03

**Authors:** Jung Ryul Kim, Young Jae Moon, Keun Sang Kwon, Jun Sang Bae, Sajeev Wagle, Taek Kyun Yu, Kyoung Min Kim, Ho Sung Park, Ju-Hyung Lee, Woo Sung Moon, Ho Lee, Myoung Ja Chung, Kyu Yun Jang

**Affiliations:** 1 Department of Orthopaedic Surgery, Chonbuk National University Medical School, Research Institute of Clinical Medicine and Research Institute for Endocrine Sciences, Jeonju, Republic of Korea; 2 Department of Preventive Medicine, Chonbuk National University Medical School, Research Institute of Clinical Medicine and Research Institute for Endocrine Sciences, Jeonju, Republic of Korea; 3 Department of Pathology, Chonbuk National University Medical School, Research Institute of Clinical Medicine and Research Institute for Endocrine Sciences, Jeonju, Republic of Korea; 4 Department of Forensic Medicine, Chonbuk National University Medical School, Research Institute of Clinical Medicine and Research Institute for Endocrine Sciences, Jeonju, Republic of Korea; Josep Carreras Leukaemia Research Institute, University of Barcelona, Spain

## Abstract

Recently, the roles of SIRT1 and deleted in breast cancer 1 (DBC1) in human cancer have been extensively studied and it has been demonstrated that they are involved in many human carcinomas. However, their clinical significance for soft-tissue sarcomas has not been examined. In this study, we evaluated the expression and prognostic significance of the expression of SIRT1, DBC1, P53, β-catenin, cyclin D1, and KI67 in 104 cases of soft-tissue sarcomas. *RESULTS:* Immunohistochemical expression of SIRT1, DBC1, P53, β-catenin, and cyclin D1 were seen in 71%, 74%, 53%, 48%, and 73% of sarcomas, respectively. The expression of SIRT1, DBC1, P53, β-catenin, and cyclin D1 were significantly correlated with advanced clinicopathological parameters such as higher clinical stage, higher histological grade, increased mitotic counts, and distant metastasis. The expression of SIRT1, DBC1, P53, β-catenin, cyclin D1, and KI67 were significantly correlated with each other and positive expression of all of these predicted shorter overall survival and event-free survival by univariate analysis. Multivariate analysis revealed the expression of SIRT1 as an independent prognostic indicator for overall survival and event-free survival of sarcoma patients. In conclusion, this study demonstrates that SIRT1- and DBC1-related pathways may be involved in the progression of soft-tissue sarcomas and can be used as clinically significant prognostic indicators for sarcoma patients. Moreover, the SIRT1- and DBC1-related pathways could be new therapeutic targets for the treatment of sarcomas.

## Introduction

SIRT1 (silent mating type information regulation 2 homolog 1) is a type III histone deacetylase, but, also deacetylates non-histone proteins, especially proteins involved in tumorigenesis [1]–[4]. A role of SIRT1 as a non-histone deacetylase tumor promoter which is centrally mediated by functional inhibition of P53 has been proposed [1]. Recent extensive studies have shown that changes in SIRT1-mediated signaling give survival benefits under the stress conditions, which is closely related with tumorigenesis [1], [3]–[7]. The expression of SIRT1 increases resistance to anticancer agents [8], [9] and is associated with progression of cancers and poor prognosis of cancer patients [3], [5], [10], [11]. SIRT1 was determined to be an indicator of poor prognostic for gastric carcinoma [5], hepatocellular carcinoma [3], breast carcinoma [11], and diffuse large B cell lymphoma [10]. In addition to the role of SIRT1 as a deacetylase, recent reports have shown that SIRT1 is also involved in the transcriptional expression of various oncogenes such as c-Myc, β-catenin, cyclin D1, and survivin [3], [6], [7]. Moreover, functional inhibition of SIRT1 with nicotinamide decreased tumorigenesis in c-Myc driving liver cancer animal models [3].

Deleted in breast cancer 1(DBC1) was first identified by its deletion in breast cancer [12] and was suggested as a tumor suppressor because it acts as a suppressor of SIRT1 [10]. However, increasing recent evidence has demonstrated that DBC1 could act as tumor promoter via various signaling pathways [13]–[15]. DBC1 can act as a co-activator of hormone receptors [16] and inhibits tumor suppressors BRCA1 [13] and SUV39H1 methyltransferase [15]. In human cancers, the expression of DBC1 is associated with advanced cancer and predicted poor survival of various human malignant tumors [5], [11], [14], [17].

Most soft-tissue tumors are benign and soft-tissue sarcomas are rare. Benign soft-tissue tumors are 100 times more frequent than soft-tissue sarcomas [18]. Soft-tissue sarcomas account for less than 1% of human malignant tumors. However, there are more than 50 histological subtypes, and they show aggressive behavior [18]. Therefore, diagnosing and treating soft-tissue sarcomas are challenging to clinicians, and there is a need for new therapeutic target for the treatment of sarcoma. When considering the extensive studies and important role of SIRT1 and DBC1 in human carcinomas, there is a rationale that SIRT1 and DBC1 also could be involved in the pathogenesis of sarcoma. Recently, substantial expression of SIRT1 in soft-tissue neoplasms with myoid differentiation has been reported [19]. However, there have been no previous reports examining the prognostic significance of the expression of SIRT1 and DBC1 in soft-tissue sarcoma. Therefore, we investigated the prevalence and prognostic significance of SIRT1 and DBC1 expression in soft-tissue sarcoma patients. In addition, we investigated the expression of β-catenin and cyclin D1 expression because of both of them have been suggested as a down-stream targets of SIRT1 [3].

## Results

### Association of SIRT1, DBC1, P53, β-catenin, and cyclin D1 expression with clinicopathological characteristics of soft tissue sarcoma patients

**Figure 1:**
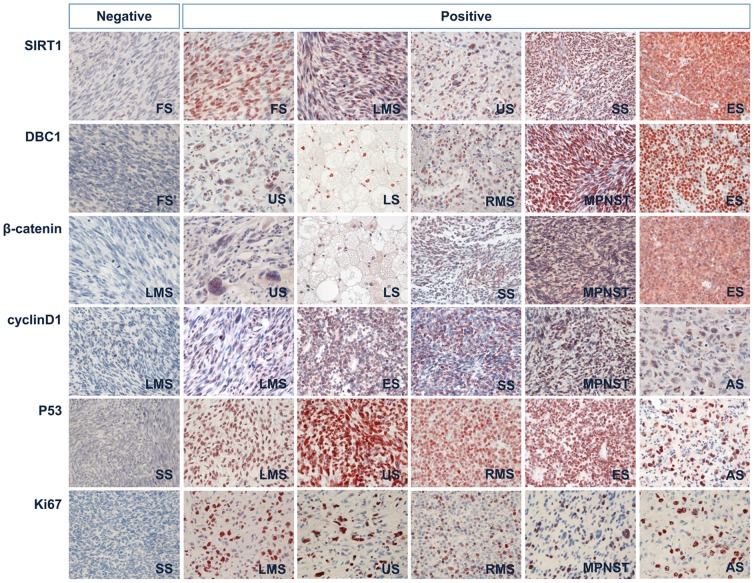
Immunohistochemical expression of SIRT1, DBC1, β-catenin, cyclin D1, P53, and Ki67 in various soft tissue sarcomas. All markers are expressed primarily in the nuclei of the tumor cells. Abbreviations: FS, adult fibrosarcoma; LMS, leiomyosarcoma; US, undifferentiated sarcoma; SS, synovial sarcoma; ES, Ewing sarcoma; LS, liposarcoma; RMS, rhabdomyosarcoma; MPNST, malignant peripheral nerve sheath tumor; AS, angiosarcoma. Original magnification, x400.

The variable clinicopathological features of sarcoma are summarized in Table 1. As shown in Figure 1, the expression of SIRT1, DBC1, P53, cyclin D1, and Ki67 were primarily in the nuclei. Cytoplasmic expression of SIRT1 was seen in some cases. Although β-catenin is expressed in the cytoplasmic membrane, cytoplasm, and nuclei, we evaluated nuclear β-catenin expression only. Positive expression of SIRT1, DBC1, P53, β-catenin, and cyclin D1 were seen in 71% (74 of 104), 74% (77 of 104), 53% (55 of 104), 48% (50 of 104), and 73% (76 of 104) of sarcomas, respectively. The expression of these markers according to the histological type of soft-tissue sarcomas was shown in Table 1. Expression of SIRT1 significantly correlated with tumor stage (*P* = 0.013), distant metastasis (*P* = 0.001), histological grade (*P* = 0.008), mitotic count (*P* = 0.002), Ki67 index (*P* = 0.014), cyclin D1 expression (*P*<0.001), β-catenin expression (*P*<0.001), P53 expression (*P* = 0.003), and DBC1 expression (*P*<0.001). DBC1 expression was also significantly correlated with tumor stage (*P* = 0.019), distant metastasis (*P* = 0.003), histological grade (*P* = 0.013), mitotic count (*P* = 0.032), cyclin D1 expression (*P*<0.001), β-catenin expression (*P*<0.001), and P53 expression (*P* = 0.005). P53 expression significantly correlated with patient age, tumor stage, distant metastasis, histological grade, tumor differentiation, mitotic count, Ki67 index, cyclin D1 expression, and β-catenin expression. The expression of β-catenin was significantly associated with histological grade, tumor differentiation, mitotic count, and cyclin D1 expression. The expression of cyclin D1 was significantly associated with tumor stage, histological grade, tumor differentiation, and mitotic count. Ki67 index was significantly associated with tumor stage, distant metastasis, histological grade, tumor necrosis, and mitotic count (Table 2).

**Table 1:** The expression of SIRT1, DBC1, P53, β-catenin, cyclin D1, and Ki67 according to the histological type of soft-tissue sarcomas.

Histological type	Total	SIRT1 +	DBC1 +	P53 +	β-catenin +	cyclin D1 +	Ki67 index +
Leiomyosarcoma	20	13 (65%)	16 (80%)	13 (65%)	5 (25%)	16 (80%)	11 (55%)
Synovial sarcoma	16	13 (81%)	13 (81%)	9 (56%)	14 (88%)	14 (88%)	4 (25%)
Undifferentiated sarcoma	11	9 (82%)	10 (91%)	9 (82%)	7 (64%)	10 (91%)	10 (91%)
Myxoid liposarcoma	10	4 (40%)	4 (40%)	3 (30%)	1 (10%)	5 (50%)	6 (60%)
Well differentiated liposarcoma	4	3 (75%)	4 (100%)	0 (0%)	1 (25%)	2 (50%)	1 (25%)
Dedifferentiated liposarcoma	3	0 (0%)	0 (0%)	1 (33%)	0 (0%)	0 (0%)	1 (33%)
Ewing sarcoma	6	5 (83%)	4 (67%)	4 967%)	4 (67%)	5 (83%)	5 (83%)
Malignant peripheral nerve sheath tumor	6	6 (100%)	4 (67%)	1 (17%)	3 (50%)	4 (67%)	3 (50%)
Adult fibrosarcoma	5	4 (80%)	4 (80%)	1 (20%)	3 (60%)	3 (60%)	2 (40%)
Angiosarcoma	5	5 (100%)	4 (80%)	4 (80%)	4 (80%)	4 (80%)	5 (100%)
Myxofibrosarcoma	4	1 (25%)	2 (50%)	2 (50%)	1 (25%)	2 (50%)	3 (75%)
Epitheliod sarcoma	4	3 (75%)	3 (75%)	3 (75%)	1 (25%)	2 (50%)	4 (100%)
Alveolar rhabdomyosarcoma	3	3 (100%)	3 (100%)	2 (67%)	2 (67%)	3 (100%)	3 (100%)
Embryonal rhabdomyosarcoma	2	1 (50%)	1 (50%)	1 (50%)	0 (0%)	2 (100%)	1 (50%)
Pleomorphic rhabdomyosarcoma	2	2 (100%)	2 (100%)	1(50%)	2 (100%)	2 (100%)	1 (50%)
Low grade myofibroblastic sarcoma	2	1 (50%)	2 (100%)	1 (50%)	1 (50%)	1 (50%)	1 (50%)
Clear cell sarcoma	1	1 (100%)	1 (100%)	0 (0%)	1 (100%)	1 (100%)	0 (0%)

**Table 2:** Clinicopathological variables and the expression status of SIRT1, DBC1, P53, β-catenin, cyclin D1, and Ki67 in soft tissue sarcomas.

Characteristics		*N*	SIRT1		DBC1		P53		β-catenin		cyclin D1		Ki67 index	
			positive	*P*	positive	*P*	positive	*P*	positive	*P*	positive	*P*	> 10/5 HPF	*P*
Age, y	< 60	67	44 (66%)	0.097	49 (73%)	0.777	30 (45%)	0.026	30 (45%)	0.365	48 (72%)	0.657	35 (52%)	0.074
	≥ 60	37	30 (81%)		28 (76%)		25 (68%)		20 (45%)		28 (76%)		26 (70%)	
Sex	female	45	22 (60%)	0.187	31 (69%)	0.296	22 (49%)	0.476	19 (42%)	0.297	36 (80%)	0.165	25 (56%)	0.575
	male	59	45 (76%)		46 (78%)		33 (56%)		31 (53%)		40 (68%)		36 (61%)	
Stage	I and II	53	33 (62%)	0.013	34 (64%)	0.019	17 (32%)	< 0.001	24 (45%)	0.561	34 (64%)	0.036	23 (43%)	0.001
	III and IV	51	42 (82%)		43 (84%)		38 (75%)		26 (51%)		42 (82%)		38 (75%)	
Depth	superficial	39	24 (62%)	0.094	26 (67%)	0.184	17 (44%)	0.141	17 (44%)	0.478	27 (69%)	0.493	23 (59%)	0.959
	deep	65	50 (77%)		51 (78%)		38 (58%)		33 (51%)		49 (75%)		38 (58%)	
Tumor size, cm	≤ 5	33	22 (67%)	0.491	26 (79%)	0.451	17 (52%)	0.849	16 (48%)	0.955	23 (70%)	0.596	21 (64%)	0.482
	> 5	71	52 (73%)		51 (72%)		38 (54%)		34 (48%)		53 (75%)		40 (56%)	
LN metastasis	absence	89	62 (70%)	0.414	67 (75%)	0.481	45 (51%)	0.248	43 (48%)	0.906	64 (72%)	0.513	52 (58%)	0.909
	presence	15	12 (80%)		10 (67%)		10 (67%)		7 (47%)		12 (80%)		9 (60%)	
Distant metastasis	absence	73	45 (62%)	0.001	48 (66%)	0.003	31 (42%)	0.001	32 (44%)	0.184	51 (70%)	0.257	38 (52%)	0.036
	presence	31	29 (94%)		29 (94%)		24 (77%)		18 (58%)		25 (81%)		23 (74%)	
Histological Grade	1	22	10 (45%)	0.008	13 (59%)	0.013	4 (18%)	< 0.001	6 (27%)	0.006	10 (45%)	0.002	7 (32%)	< 0.001
	2	34	25 (74%)		22 (65%)		15 (44%)		13 (38%)		25 (74%)		17 (50%)	
	3	48	39 (81%)		42 (88%)		36 (75%)		31 (65%)		41 (85%)		37 (77%)	
Tumor necrosis	no necrosis	48	29 (60%)	0.072	31 (65%)	0.118	20 (42%)	0.078	24 (50%)	0.284	30 (63%)	0.075	22 (46%)	0.036
	< 50%	42	33 (79%)		34 (81%)		25 (60%)		17 (40%)		34 (81%)		28 (67%)	
	≥ 50%	14	12 (86%)		12 (86%)		10 (71%)		9 (64%)		12 (86%)		11 (79%)	
Tumor differentiation	1	9	5 (56%)	0.209	7 (78%)	0.249	1 (11%)	0.005	1 (11%)	< 0.001	5 (56%)	0.033	2 (22%)	0.063
	2	40	26 (65%)		26 (65%)		18 (45%)		13 (33%)		25 (63%)		24 (60%)	
	3	55	43 (78%)		44 (80%)		36 (65%)		36 (65%)		46 (84%)		35 (64%)	
Mitotic count	0–9/10 HPF	40	21 (53%)	0.002	24 (60%)	0.032	12 (30%)	< 0.001	12 (30%)	0.014	22 (55%)	0.003	15 (38%)	0.002
	10–19/10 HPF	22	20 (91%)		19 (86%)		11 (50%)		13 (59%)		20 (91%)		15 (68%)	
	> 19/10 HPF	42	33 (79%)		34 (81%)		32 (76%)		25 (60%)		34 (81%)		31 (74%)	
Ki67 index	≤ 10/5HPF	43	25 (58%)	0.014	29 (67%)	0.198	8 (19%)	< 0.001	19 (44%)	0.505	28 (65%)	0.124		
	> 10/5HPF	61	49 (80%)		48 (79%)		47 (77%)		31 (51%)		48 (79%)			
cyclin D1	negative	28	10 (36%)	< 0.001	9 (32%)	< 0.001	8 (29%)	0.003	4 (14%)	< 0.001				
	positive	76	64 (84%)		68 (89%)		47 (62%)		46 (61%)					
β-catenin	negative	54	28 (52%)	< 0.001	30 (56%)	< 0.001	20 (37%)	< 0.001						
	positive	50	46 (92%)		47 (94%)		35 (70%)							
P53	negative	49	28 (57%)	0.003	30 (61%)	0.005								
	positive	55	46 (84%)		47 (85%)									
DBC1	negative	27	8 (30%)	< 0.001										
	positive	77	66 (86%)											

Abbreviations: SIRT1, silent mating-type information regulation 2 homologue 1; DBC1, deleted in breast cancer 1; HPF, high-power fields; LN, lymph node.

### The expression of SIRT1, DBC1, P53, β-catenin, and cyclin D1, and Ki67 index in sarcomas correlate with reduced overall survival and event-free survival

**Figure 2:**
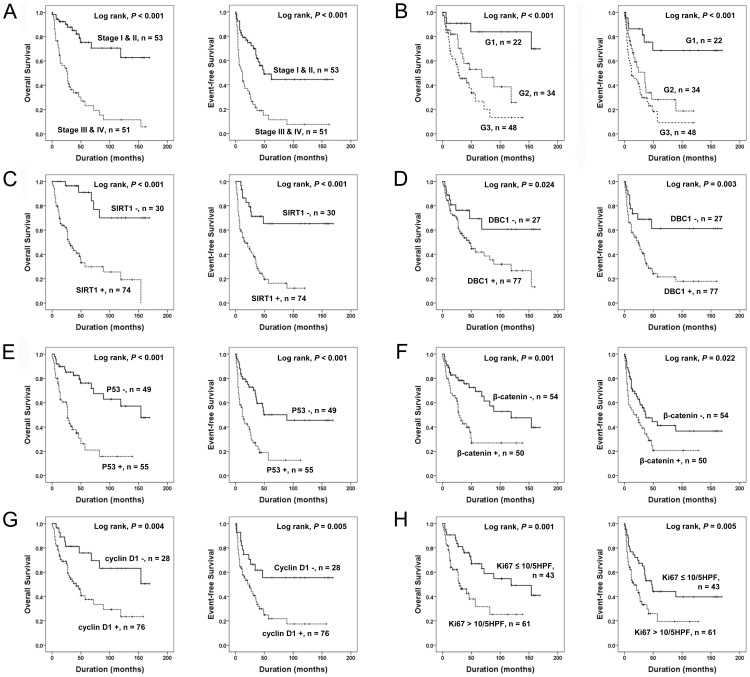
Kaplan-Meier survival analysis of soft tissue sarcoma patients. Overall survival and event-free survival according to tumor stage (A), histological grade (B), and the expression of SIRT1 (C), DBC1 (D), P53 (E), β-catenin (F), cyclin D1 (G), and Ki67 (H).

Univariate Cox regression analysis for OS and EFS are shown in Table 3 and Kaplan-Meier survival curves for the impact to OS and EFS are shown in Figure 2. Older age of patients, high tumor stage, high histological grade, deeply located tumor, presence of tumor necrosis, increased mitotic count, and presence of distant metastasis predicted shorter OS and EFS (Figure 2 A and B). Expression of SIRT1 was significantly associated with shorter OS [*P*<0.001, HR; 7.357, 95% confidence interval (95% CI); 2.871–18.855] and EFS (*P*<0.001, HR; 4.186, 95% CI; 2.055–8.525) by univariate analysis (Figure 2 C). DBC1 expression was also significantly associated with shorter OS (*P* = 0.029, HR; 2.338, 95% CI; 1.090–5.013) and EFS (*P* = 0.005, HR; 2.761, 95% CI; 1.361–5.601) by univariate analysis (Figure 2 D). The expression of P53, β-catenin, and cyclin D1 were significantly associated with shorter OS (*P*<0.001, *P* = 0.002, and *P* = 0.006, respectively) and EFS (*P*<0.001, *P* = 0.026, and *P* = 0.007, respectively) by univariate analysis (Figure 2 E F and G). The Ki67 index also predicted shorter OS (*P* = 0.002) and EFS (*P* = 0.007) (Figure 2 H).

**Table 3:** Univariate Cox regression analysis for overall survival and event-free survival in soft tissue sarcoma patients.

Characteristics	*N*	OS		EFS	
		HR (95% CI)	*P*	HR (95% CI)	*P*
Age, y, ≥ 60 (*vs* < 60)	37/104	1.961 (1.129–3.405)	0.017	2.073 (1.261–3.408)	0.004
Sex, male (*vs* female)	59/104	1.290 (0.737–2.256)	0.373	1.201 (0.731–1.974)	0.470
Stage, III and IV (*vs* I and II)	51/104	5.400 (2.809–10.381)	< 0.001	3.747 (2.205–6.367)	< 0.001
Depth, deep (*vs* superficial)	65/104	3.735 (1.754–7.951)	< 0.001	2.923 (1.614–5.296)	< 0.001
Tumor size, > 5 cm (*vs* ≤ 5 cm)	71/104	1.611 (0.856–3.029)	0.139	1.147 (0.675–1.947)	0.612
LN metastasis, presence (*vs* absence)	15/104	2.224 (1.138–4.345)	0.019	1.843 (0.982–3.460)	0.057
Distant metastasis, presence (*vs* absence)	31/104	4.264 (2.433–7.472)	< 0.001	4.953 (2.946–8.329)	< 0.001
Histological Grade, 1	22/104	Ref	< 0.001	Ref	< 0.001
2	34/104	5.058 (1.467–17.437)	0.010	3.648 (1.460–9.110)	0.006
3	48/104	8.866 (2.670–29.442)	<0.001	5.468 (2.272–13.161)	< 0.001
Tumor necrosis, no necrosis	48/104	Ref	<0.001	Ref	0.004
< 50%	42/104	3.464 (1.794–6.687)	<0.001	2.481 (1.431–4.303)	0.001
≥ 50%	14/104	4.050 (1.767–9.283)	<0.001	2.318 (1.092–4.924)	0.029
Tumor differentiation, 1	8/104	Ref	0.021	Ref	0.090
2	40/104	1.861 (0.540–6.416)	0.325	1.754 (0.603–5.099)	0.302
3	55/104	3.670 (1.102–12.221)	0.034	2.649 (0.940–7.459)	0.065
Mitotic count, 0–9/10 HPF	40/104	Ref	0.002	Ref	< 0.001
10–19/10 HPF	22/104	3.572 (1.574–8.104)	0.002	3.261 (1.617–6.575)	< 0.001
> 19/10 HPF	42/104	3.589 (1.717–7.499)	< 0.001	2.993 (1.603–5.589)	< 0.001
SIRT1, positive (*vs* negative)	74/104	7.357 (2.871–18.855)	< 0.001	4.186 (2.055–8.525)	< 0.001
DBC1, positive (*vs* negative)	77/104	2.338 (1.090–5.013)	0.029	2.761 (1.361–5.601)	0.005
P53, positive (*vs* negative)	55/104	4.303 (2.260–8.195)	< 0.001	3.049 (1.790–5.195)	< 0.001
β-catenin, positive (*vs* negative)	50/104	2.556 (1.423–4.591)	0.002	1.760 (1.071–2.894)	0.026
cyclin D1, positive (*vs* negative)	76/104	2.811 (1.342–5.888)	0.006	2.440 (1.269–4.689)	0.007
Ki67 index, > 10/5 HPF (*vs* ≤ 10/5 HPF)	61/104	2.606 (1.418–4.790)	0.002	2.060 (1.219–3.480)	0.007

Abbreviations: OS, overall survival; EFS, event-free survival; HR, hazard ratio; 95% CI, 95% confidence interval; HPF, high-power fields; LN, lymph node; SIRT1, silent mating-type information regulation 2 homologue 1; DBC1, deleted in breast cancer 1; Ref, reference.

### The expression of SIRT1 in soft-tissue sarcoma is an independent prognostic factor for shorter event-free survival and poor overall survival

Multivariate analysis was performed using the variables significantly correlated with OS or RFS by univariate Cox regression analysis. The variables considered in the multivariate analysis for OS and RFS were the age of the patients, tumor stage, tumor depth, lymph node metastasis, distant metastasis, histological grade, tumor necrosis, tumor differentiation, mitotic count, Ki67 index, and the expression of SIRT1, DBC1, P53, β-catenin, and cyclin D1. From the multivariate analysis, the expression of SIRT1 was an independent prognostic indicator significantly associated with both OS and EFS. The patients with SIRT1 expression had a 10.062-fold (95% CI, 2.851–35.509) greater risk of death (*P*<0.001) and a 2.459-fold (95% CI, 1.166–5.185) greater risk of EFS (*P* = 0.018). In addition, tumor stage (*P* = 0.002), tumor depth (*P* = 0.007), tumor necrosis (*P* = 0.007), P53 expression (*P* = 0.033), DBC1 expression (*P*<0.001), and β-catenin expression (*P* = 0.020) were independent prognostic indicators of shorter OS by multivariate analysis. Tumor depth (*P* = 0.017), distant metastasis (*P*<0.001), tumor necrosis (*P* = 0.035), and P53 expression (*P* = 0.004) were independent prognostic indicators of EFS (Table 4).

**Table 4:** Multivariate Cox regression analysis for overall survival and event-free survival in soft tissue sarcoma patients.

Characteristics	OS		EFS	
	HR (95% CI)	*P*	HR (95% CI)	*P*
SIRT1, positive (*vs* negative)	10.062 (2.851–35.509)	<0.001	2.459 (1.166–5.185)	0.018
P53, positive (*vs* negative)	2.412 (1.074–5.421)	0.033	2.265 (1.298–3.952)	0.004
DBC1, positive (*vs* negative)	6.501 (2.160–19.565)	<0.001		
β-catenin, positive (*vs* negative)	2.491 (1.158–5.361)	0.020		
Stage, III and IV (*vs* I and II)	3.424 (1.547–7.579)	0.002		
Depth, deep (*vs* superficial)	2.927 (1.347–6.357)	0.007	2.128 (1.142–3.965)	0.017
Tumor necrosis, no necrosis	Ref	0.007	Ref	0.035
< 50%	3.163 (1.507–6.641)	0.002	1.785 (1.019–3.126)	0.043
≥ 50%	1.566 (0.651–3.767)	0.317	0.799 (0.355–1.796)	0.587
Distant metastasis, presence (*vs* absence)			3.263 (1.827–5.827)	< 0.001

Abbreviations: OS, overall survival; EFS, event-free survival; HR, hazard ratio; 95% CI, 95% confidence interval; SIRT1, silent mating-type information regulation 2 homologue 1; DBC1, deleted in breast cancer 1; HPF, high-power fields; Ref, reference.

## Discussion

In this study we have shown that SIRT1, DBC1, P53, β-catenin, and cyclin D1 were expressed in 71%, 74%, 53%, 48%, and 73% of human soft-tissue sarcomas, respectively, and they significantly correlated with advanced clinicopathological parameters such as higher clinical stage, higher histological grade, poorly differentiation of sarcoma, increased mitotic counts, and distant metastasis. Moreover, the expression of SIRT1, DBC1, P53, β-catenin, cyclin D1, and KI67 were significantly correlated with each other, and their expression predicted shorter survival by univariate analysis. Especially, the expression of SIRT1 was an independent prognostic indicator of OS and EFS by multivariate analysis. These findings suggest that the expression of SIRT1 and DBC1 can be used as clinically significant prognostic indicators for sarcoma patients. Moreover, SIRT1- and DBC1-related pathways may be involved in the progression of soft-tissue sarcomas and SIRT1- and DBC1-related pathways may provide targets for novel therapeutic approaches for soft-tissue sarcomas.

The role of SIRT1 in human carcinomas has been extensively studied. However, the study for the expressional status of SIRT in human mesenchymal tumors is limited. Recently, common expression of SIRT1 in soft-tissue tumors with myoid differentiation compared with other types of soft-tissue tumor has been reported [19]. This report has shown that 29 of 49 (64%) cases of leiomyosarcoma expressed cytoplasmic SIRT1 but could not detect SIRT1 expression in 7 synovial sarcoma, 5 liposarcoma, 4 Ewing sarcoma, 4 malignant peripheral nerve sheath tumor, 4 undifferentiated pleomorphic sarcoma, and 4 clear cell sarcoma [19]. However, as shown in Figure 1 and Table 1, our result showed that the expression of SIRT1 is common in soft-tissue sarcomas regardless of histological type. This discrepancy might come from the specificity of used anti-SIRT1 antibody and evaluation for the subcellular localization of SIRT expression. Concerning the subcellular localization of SIRT1, it has been reported that SIRT1 expresses both nuclei and cytoplasm [3], [5], [10], [11], [20]. In contrast to the role of SIRT1 for the resistance for the stresses [1], [3], [4], cytoplasmic localization of SIRT sensitized the cells to oxidative stress-mediated apoptosis [20]. In addition, the prognostic effect of SIRT1 according to the expressional localization was variably reported. In gastric carcinoma, only the nuclear expression of SIRT predicted poor prognosis of patients but not in cytoplasmic expression of SIRT1 [5]. In contrast, both nuclear and cytoplasmic expression of SIRT1 associated with poor prognosis of breast carcinoma patients [11]. In our study, we evaluated nuclear expression of SIRT because main localization of SIRT1expression was nuclei as shown in Figure 1 and nuclear expression is easy to evaluate and predicted poor survival in various human malignant tumors. In this study, nuclear expression of SIRT1 was an independent prognostic indicator for OS and EFS in soft-tissue sarcoma patients. When separately analyzed the soft-tissue sarcomas according to the tumor stage (stage I and II *versus* stage III and IV) and histological grade (grade 1 *versus* grade 2 and 3), nuclear expression of SIRT1 predicted sorter OS and EFS regardless of the tumor stage or histological grade (Figure S1).

There are seven types of sirtuins (SIRT1-7). Among them, SIRT1 is known for its role in prolonging mammalian cell survival under stress [2], [4], [21]. However, its role in the resistance to the stresses suggested that SIRT1 could be involved in the progression of cancers by regulating histone and non-histone proteins [1], [3], [6], [7], [21]. In addition, other types of sirtuins could also be involved in tumorigenesis. SIRT2 stabilized Myc oncoproteins and promoted Myc-induced oncogenic effects [22], [23]. However, the roles of SIRT3 and SIRT6 in tumorigenesis are controversial. Their potential roles as tumor promoters and tumor suppressors have been suggested in various reports [24]–[26]. Recently, SIRT4 has been suggested as a tumor suppressor by regulating DNA damage response pathways [27].

Concerning to the role of SIRT1 in human malignant tumors most studies demonstrated that the expression of SIRT1 in human tissue related to the survival of cells and present benefits to the survival of cells despite some controversies [3], [5]–[7], [10], [11]. In functioning cells and tissue, SIRT1 expression provides resistance to various stresses and repairs genetic damage [1], [9], [28]. However, when there are oncogenic signals, SIRT1 served to promote the proliferation or survival of tumor cells [3], [8]. In addition, the expression of SIRT1 increased in human cancer tissue and during experimental carcinogenesis [3], [5], [11], [14], [21]. This phenomenon raised the question of whether the increased expression of SIRT1 in cancer is the cause of the cancer or the consequence of the deregulation of key factors involved in the development of cancer. The expression of SIRT1 is positively controlled by the oncogenes *c-Myc* and *N-Myc* [3], [6], [7], [29], and the function of SIRT1 is post-transcriptionally regulated by CK2-mediated phosphorylation [30] and post-transcriptionally repressed by microRNA-204 [31]. In addition, overexpression of SIRT1 induced chemoresistance of cancer cells by up-regulating P-glycoprotein expression [9]. The higher expression of SIRT1 in chemoresistant types of cancer cells raises the possibility that the increased expression of SIRT1 in the poor prognostic group of cancer is the consequence of the progression of cancer. However, ectopic expression of SIRT1 increases the proliferation of cancer cells and blocks stress-induced apoptosis [3], [4], [8], [9]. Especially, SIRT1 forms a positive feedback loop with the oncogenes *c-Myc* and *N-Myc* [3], [6], [7], [29]. In addition, SIRT1 induces expression of tumor progressing targets such as constitutive Wnt signaling pathway and survivin [3], [32]. Furthermore, inhibition of SIRT1 inhibited the proliferation of cancer cells and triggered cancer cell death [33]–[36]. Moreover, SIRT1 mediated cellular proliferation was cancer specific. Knock-down of SIRT1 enhanced apoptosis only in the cancer cells, but not in normal cells [37]. The possibility that SIRT1 could be a therapeutic target of human cancer has also been suggested in xenograft tumorigenic assays. The SIRT1 inhibitor amurensin G increased doxorubicin responsiveness in MCF-7 cells [38]. In addition, SIRT1 inhibitor nicotinamide delayed tumor initiation in c-Myc mediated liver-specific tumorigenesis in a murine model [3].

In human cancers, SIRT1-mediated resistance to death closely related with deacetylation-mediated inhibition of death-related proteins such as P53 and FoxO3 [1], [4]. Especially, the expression of both SIRT1 and P53 were closely related with progression of cancers and poor prognosis of cancer patients [3], [5], [10], [11]. In this study, the expression of SIRT1 and P53 were significantly correlated. In addition, both of them predicted poor survival of sarcoma patients and were closely related with advanced clinicopathological indicators of soft-tissue sarcomas. Although the prognostic significance of P53 expression in soft-tissue sarcomas is well-known [39], [40], this study is the first to demonstrate SIRT1 as a prognostic indicator of soft-tissue sarcomas. In addition, our study suggests that SIRT1- and P53-related pathways may also have roles in the tumorigenesis of soft-tissue sarcoma. However, immunohistochemical identification of P53 may not directly represent the functional status of P53, especially without knowing mutational status of the *TP53* gene. Therefore, further study is needed to explore the exact mechanism of SIRT1- and P53-related tumorigenesis of sarcoma.

The Wnt/β-catenin signaling pathway is critical to the survival and proliferation of cells [41]–[44]. When the Wnt protein is activated, β-catenin dissociates from the destructive complex and translocates to the nuclei. In the nuclei, β-catenin binds to TCF and induces downstream signaling that is involved in the proliferation of cells [45]. Although, there are some controversies [3], most studies have shown that nuclear expression of β-catenin is associated with the progression of human cancers. In human sarcomas, nuclear expression of β-catenin predicted poor prognosis of synovial sarcoma [46], [47]. Our results have also indicated that the expression of β-catenin and cyclin D1 are significantly associated with shorter OS and EFS by univariate analysis. Concerning the role of SIRT1, in addition to the role of SIRT1 as an epigenetic acetylation modifier, SIRT1 could induce the expression of various oncogenes and *vice versa*. The expression of SIRT1 was reversibly controlled by the expressional status of oncogene *c-Myc* [3], [6], [7]. SIRT1 also induces the transcription of c-Myc, β-catenin and the down-stream cyclin D1, and survivin [3]. This study has also demonstrated a significant correlation between the expression of SIRT1 and β-catenin, in addition to the prognostic role of SIRT1 in soft-tissue sarcomas. Therefore, when considering the signaling relationship between SIRT1 and β-catenin in carcinoma [3] and a possible relationship in sarcoma, our results suggest that SIRT1- and β-catenin-related signaling may be involved in both carcinomas and sarcomas, and SIRT1- and β-catenin-related signaling could be therapeutic targets for the treatment of soft-tissue sarcomas.

In this study, the pro-proliferative role of SIRT1 and β-catenin in sarcoma is supported by significant correlations of their expression with higher mitotic count and Ki67 index. The mean Ki67 index of SIRT1-expressing sarcomas was eight times higher than SIRT1-negative sarcomas (mean ± standard error: 434 ± 85 *versus* 59 ± 24, 2-tailed *t*-test; *P* = *0.*006). The sarcomas expressing β-catenin or cyclin D1 also had a significantly higher Ki67 index (2-tailed *t*-test; *P* = 0.021 and *P* = 0.014, respectively). A positive correlation of SIRT1 expression and Ki67 index has also been reported in liver cancer and the expression level of SIRT1 was directly correlated with the proliferative potential of tumor cells [3]. In addition, Ki67 index itself was predictive for OS and EFS of soft-tissue sarcomas. In agreement with our findings, Ki67 as a prognostic indicator of soft-tissue sarcomas has been reported in the soft-tissue sarcoma [39], [48] and malignant fibrous histiocytoma [49].

In this report, we are the first to demonstrate that DBC1 expression in soft-tissue sarcoma significantly correlated with higher tumor stage, higher histological grade, presence of distant metastasis, and increased mitotic count. Moreover, DBC1 expression predicted shorter OS and EFS. In line with our results, DBC1 expression significantly correlated with the progression and survival of human carcinomas, such as gastric carcinoma [5], breast carcinoma [11], esophageal carcinoma [14], and diffuse large B cell lymphoma [17]. Although DBC1 was first recognized as a tumor suppressor because it is deleted in breast cancer [12] and principally inhibits SIRT1 [10], recently there has been increasing evidence that DBC1 has an important role in the progression of human cancers via various cellular pathways [13], [15]. In addition, co-expression of DBC1 and SIRT1 in human cancers is becoming a more common phenomenon, as presented in hepatocellular carcinoma [50], gastric carcinoma [5], and breast carcinoma [11]. Our result also has shown that the expression of DBC1 and SIRT1 are positively correlated and both closely related with poor prognosis of sarcoma. These findings raised the possibility that increased expression of DBC1 in advanced cancer could be a consequence of tumor progression. However, recent evidence has shown that DBC1 has its own role in the progression of human cancers by inhibiting the tumor suppressors BRCA1 [13] and SUV39H1 methyltransferase [15], and is involved in the regulation of androgen receptor [16] and estrogen receptor α [51]. Recent report has shown that DBC1 inhibit anoikis by activating the NF-κB pathway [52]. In our study, the expression of DBC1 was significantly correlated with the expression of β-catenin, cyclin D1, and P53. Therefore, our results suggest that DBC1 may also be involved in the development and progression of sarcoma in conjunction with various oncogenic signals.

In breast cancer patients, DBC1 expression was associated with shorter survival in the subpopulation who received adjuvant chemotherapy and/or endocrine therapy [11]. In addition, depletion of DBC1 increased hormone-independent apoptosis of breast cancer cells [53] and inhibited proliferation and invasion of esophageal cancer cells [14]. Therefore, DBC1 inhibition in combination with conventional anti-cancer therapy might be effective. Especially, in situations where the DBC1-SIRT1 interaction is weak, the depletion of DBC1 induced breast cancer cell death in response to ultraviolet irradiation [54]. Accordingly, the application of DBC1-targetted therapy could be applicable in cancers where the SIRT1-DBC1 interaction has been deregulated. However, previous studies which have examined the use of DBC1 as a therapeutic target of human cancer have been limited. Therefore further study is needed and we suggest that DBC1-targeted therapy may also be applicable to the treatment of the unfavorable group of sarcoma expressing DBC1.

In conclusion, this study is the first to demonstrate that the expression of SIRT1 and DBC1 could be used as novel prognostic indicators of soft-tissue sarcoma. In addition, SIRT1, β-catenin, and DBC1-related pathways may be involved in the progression of sarcomas and could be new therapeutic targets for the treatment of soft-tissue sarcomas. However, the soft-tissue sarcomas included in this study were heterogeneous. Therefore, further study focused on specific types of soft-tissue sarcoma is needed to understand the exact role of SIRT1- and DBC1-related pathways in sarcomas and determine the best use of them as therapeutic targets for the treatment of specific types of soft-tissue sarcoma.

## Materials and Methods

### Ethics

This study obtained institutional review board approval from Chonbuk National University Hospital. Written informed consent was provided according to the Declaration of Helsinki.

### Patients and samples

One hundred forty-seven cases of soft-tissue sarcoma patients who underwent curative surgical resection in Chonbuk National University Hospital between July 1998 and December 2011 were included in the present study. However, original H&E slides, paraffin-embedded tissue blocks, or clinical information were not available in thirty-six cases. All of histological types of tumor and histologic grading were retrospectively reviewed in the remaining one hundred eleven cases according to the 2013 World Health Organization classification of tumors of soft tissue and bone [18]. Among the eleven well differentiated liposarcoma, seven cases were excluded in this study because these cases were atypical lipomatous tumor according to the 2013 World Health Organization classification of tumors of soft tissue and bone [18]. Four cases of well differentiated liposarcoma developed in retroperitonium were included in this study. Therefore, 104 cases of soft-tissue sarcoma were finally included in this study. Clinical information was obtained by reviewing medical records. Forty-one patients received adjuvant chemotherapy, thirty-four patients received radiation therapy, sixteen received both adjuvant chemotherapy and radiation therapy, and forty-five patients received no adjuvant treatment. The sarcomas included in this study according to the histological types were 20 leiomyosarcoma, 16 synovial sarcoma, 11 undifferentiated sarcoma, 10 myxoid liposarcoma, 4 well differentiated liposarcoma, 3 dedifferentiated liposarcoma, 6 Ewing sarcoma, 6 malignant peripheral nerve sheath tumor, 5 adult fibrosarcoma, 5 angiosarcoma, 4 myxofibrosarcoma, 4 epithelioid sarcoma, 3 alveolar rhabdomyosarcoma, 2 embryonal rhabdomyosarcoma, 2 pleomorphic rhabdomyosarcoma, 2 low grade myofibroblastic sarcoma, and one clear cell sarcoma. Histological grading was performed according to the FNCLCC (French Fédération Nationale des Centres de Lutte Contre le Cancer) system [18]. Staging of soft-tissue sarcoma was based on both histological and clinical information according to the guidelines of the tumor, node, and metastasis staging system of the American Joint Committee on Cancer [55]. The patients were grouped according to their sex, age (< 60 years *versus* ≥ 60 years), tumor stage (I and II *versus* III and IV), depth of tumor (superficial *versus* deep), tumor size (≤ 5 cm *versus* > 5 cm), histological grade, tumor necrosis, tumor differentiation, mitotic count, and the presence of lymph node metastasis or distant metastasis.

### Establishment of tissue microarray and immunohistochemical staining

Tissue microarray was established from the most representative solid area of tumor from the paraffin-embedded tissue blocks after review of original H&E slides. The size of the tissue cores was 3.0 mm and one core per case was isolated from the area of highest histological grade. Immunohistochemical staining was performed on 4 µm thick sections of tissue microarray slides. The antigen retrieval procedure in sodium citrate buffer was performed after deparaffinization. Antibodies used were: SIRT1 (1∶50, Santa Cruz Biotechnology, clone H-300, CA, USA), DBC1 (1∶100, Bethyl Laboratories, Mongomery, TX, USA), P53 (1∶50, Novocastra, clone DO-7, Newcastle, UK), β-catenin (1∶100, BD Transduction Laboratories, clone 14/Beta-Catenin, CA, USA), cyclin D1 (1∶100, Cell Signaling Technology, clone 92G2, MA, USA), and Ki67 (1∶100, DAKO, clone MIB1, Glostrup, Denmark). The precise immunohistochemical staining conditions are summarized in Table S1. Immunohistochemical scoring was performed without knowledge of the clinicopathological information under a multi-viewing microscope by two pathologists (Jang KY and Kim KM) by consensus. Immunostaining for SIRT1, DBC1, P53, β-catenin, and cyclin D1 were evaluated to estimate the nuclear positivity of tumor cells according to the Allred scoring system [56]. The nuclear staining intensity was scored as 0 (no staining), 1 (weak staining), 2 (intermediate staining), and 3 (strong staining). The area of staining was evaluated using the following sore: 0, no staining cells; 1, 1% of the cells stained positive; 2, 2–10% of the cells stained positive; 3, 11–33% of the cells stained positive; 4, 34–66% of the cells stained positive; 5, 66–100% of the cells stained positive. Thereafter, the sum of intensity score and proportion score was used for further analysis. The maximum sum score was 8 and the minimum sum score was zero. For the evaluation of immunostaining of Ki67, the number of Ki67-positive cells were counted in five high power fields (Ki67 index). Counting was performed under a Nikon ECLIPSE 50i light microscope with a 10x eyepiece with a 22 mm field of view and a 40x objective lens. The field size was 0.55 mm and the total area analyzed per case was 1.188 mm^2^.

### Statistical analysis

Immunohistochemical expression of SIRT1, DBC1, P53, β-catenin, and cyclin D1 were grouped as positive or negative by receiver operating characteristic curve analysis at the highest positive likelihood ratio point. The cut-off point for the sum score of SIRT1, DBC1, β-catenin, and cyclin D1 immunostaining was six, and that was seven for P53 staining. The immunohistochemical staining for SIRT1, DBC1, β-catenin, and cyclin D1 were scored positive when the sum score was greater than or equal to six and P53 immunostaining was scored positive when the sum score was greater than or equal to seven. Cut-off numbers for the Ki67 index were also determined by receiver operating characteristic curve analysis at the highest positive likelihood ratio point. The cut-off points were eleven Ki67-positive tumor cells in five high power fields. Pearson’s chi-square test was used to analyze the association between staining index and other clinical pathological factors. The end points of interest were overall survival (OS) and event-free survival (EFS). The follow-up end point was the date of last contact or death through October 2012. OS was measured from the date of diagnosis to the date of death or last contact. Patients who were alive at last contact were treated as censored for OS analysis. EFS was calculated as the time from diagnosis to the date of local relapse, distant metastasis, death, or last contact. Patients who were alive at last contact and who did not experience local relapse or distant metastasis were treated as censored for EFS analysis. Univariate and multivariate Cox proportional hazard regression analyses were performed to estimate the impact on OS and EFS. Kaplan-Meier survival curves were constructed to further illustrate the impact of OS and EFS where indicated. Statistical analysis performed by using SPSS software (version 18.0). *P* values less than 0.05 were considered statistically significant.

## Supporting Information

Figure S1 **Kaplan-Meier survival analysis in the subgroup of patients with soft tissue sarcoma according to the tumor stage and histological grade.** Relationship of SIRT1 expression to overall survival and event-free survival in low stage (stage I and II) (A), high stage (stage III and IV) (B), low histological grade (grade 1) (C), and high histological subgroup (grade 2 and 3) (D) subgroups.
(TIF)

Table S1 Summary of antibodies and conditions used for immunohistochemical staining.
(DOC)
