# Supplementary figures and images for: Expression of SIRT1 and DBC1 Is Associated with Poor Prognosis of Soft Tissue Sarcomas
Source: PLoS One. 2013 Sep 3;8(9):e74738. doi: 10.1371/journal.pone.0074738 (PMC3760851; doi:10.1371/journal.pone.0074738)

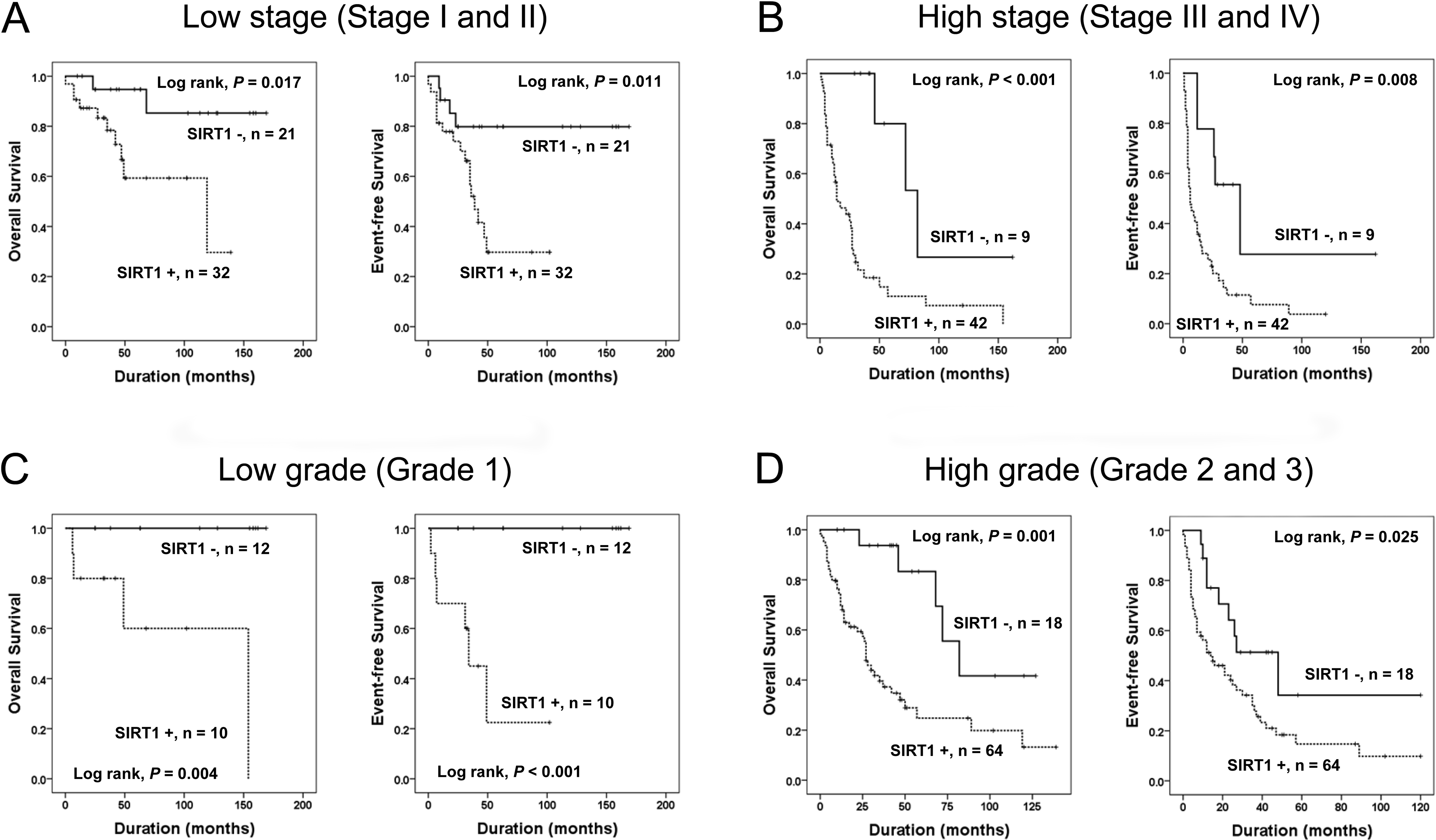

Supplement: Figure S1 — Kaplan-Meier survival analysis in the subgroup of patients with soft tissue sarcoma according to the tumor stage and histological grade. Relationship of SIRT1 expression to overall survival and event-free survival in low stage (stage I and II) (A), high stage (stage III and IV) (B), low histological grade (grade 1) (C), and high histological subgroup (grade 2 and 3) (D) subgroups. (TIF) [file pone.0074738.s001.tif]
