# Supplementary material for: Expression of SIRT1 and DBC1 Is Associated with Poor Prognosis of Soft Tissue Sarcomas
Source: PLoS One. 2013 Sep 3;8(9):e74738. doi: 10.1371/journal.pone.0074738 (PMC3760851; doi:10.1371/journal.pone.0074738)
Supplement: Table S1 — Summary of antibodies and conditions used for immunohistochemical staining. (DOC) [file pone.0074738.s002.doc]

Table S1. Summary of antibodies and conditions used for immunohistochemical staining.

| Antibody | Clone | Source | Dilution | Antigen retrieval (heat induced epitope retrieval) | IHC system | Antibody incubation |
| --- | --- | --- | --- | --- | --- | --- |
| SIRT1 | H-300 | Santa Cruz Biotechnology | 1:50 | Citrate buffer pH6.0, Microwave, 12 min | Manual, DAKO Envision* | 4℃, overnight |
| DBC1 | NA | Bethyl Laboratories | 1:100 | Citrate buffer pH6.0, Microwave, 12 min | Manual, DAKO Envision* | 4℃, overnight |
| β-catenin | 14/Beta-Catenin | BD Transduction Laboratories | 1:100 | Citrate buffer pH6.0, Microwave, 12 min | Manual, DAKO Envision* | 4℃, overnight |
| cyclin D1 | 92G2 | Cell signaling | 1:100 | Citrate buffer pH6.0, Microwave, 12 min | Manual, DAKO Envision* | 4℃, overnight |
| P53 | DO-7 | Novocastra | 1:50 | Citrate buffer pH6.0, Pressure cooker, 3.5 min | Leica BOND-X* | RT, 20 min |
| Ki67 | MIB1 | DAKO | 1:100 | Citrate buffer pH6.0, Pressure cooker, 3.5 min | Leica BOND-X* | RT, 20 min |

IHC, immunohistochemical staining; NA, not available; RT, room temperature; *, DAKO Envision detection system (DAKO, Carpinteria, CA, USA); **, automated immunohistochemistry with BOND polymer intense kit (Leica BOND-X, Leica Microsystems, Wetzlar, Germany).
